# Cellular Plasmalogen Content Does Not Influence Arachidonic Acid Levels or Distribution in Macrophages: A Role for Cytosolic Phospholipase A_2_γ in Phospholipid Remodeling

**DOI:** 10.3390/cells8080799

**Published:** 2019-07-31

**Authors:** Patricia Lebrero, Alma M. Astudillo, Julio M. Rubio, Lidia Fernández-Caballero, George Kokotos, María A. Balboa, Jesús Balsinde

**Affiliations:** 1 Instituto de Biología y Genética Molecular, Consejo Superior de Investigaciones Científicas (CSIC), Universidad de Valladolid, 47003 Valladolid, Spain; 2 Centro de Investigación Biomédica en Red de Diabetes y Enfermedades Metabólicas Asociadas (CIBERDEM), 28029 Madrid, Spain; 3 Department of Chemistry, National and Kapodistrian University of Athens, Panepistimiopolis, 15771 Athens, Greece

**Keywords:** arachidonic acid, eicosanoids, inflammation, phospholipid remodeling, phospholipase A_2_, monocytes/macrophages

## Abstract

Availability of free arachidonic acid (AA) constitutes a rate limiting factor for cellular eicosanoid synthesis. AA distributes differentially across membrane phospholipids, which is largely due to the action of coenzyme A-independent transacylase (CoA-IT), an enzyme that moves the fatty acid primarily from diacyl phospholipid species to ether-containing species, particularly the ethanolamine plasmalogens. In this work, we examined the dependence of AA remodeling on plasmalogen content using the murine macrophage cell line RAW264.7 and its plasmalogen-deficient variants RAW.12 and RAW.108. All three strains remodeled AA between phospholipids with similar magnitude and kinetics, thus demonstrating that cellular plasmalogen content does not influence the process. Cell stimulation with yeast-derived zymosan also had no effect on AA remodeling, but incubating the cells in AA-rich media markedly slowed down the process. Further, knockdown of cytosolic-group IVC phospholipase A_2_γ (cPLA_2_γ) by RNA silencing significantly reduced AA remodeling, while inhibition of other major phospholipase A_2_ forms such as cytosolic phospholipase A_2_α, calcium-independent phospholipase A_2_β, or secreted phospholipase A_2_ had no effect. These results uncover new regulatory features of CoA-IT-mediated transacylation reactions in cellular AA homeostasis and suggest a hitherto unrecognized role for cPLA_2_γ in maintaining membrane phospholipid composition via regulation of AA remodeling.

## 1. Introduction

Arachidonic acid (*cis*-5,8,11,14-eicosatetraenoic acid; AA) is the precursor of the eicosanoids, a large family of compounds with key roles in the initiation and resolution of inflammation [1]. Since AA is not found in free fatty acid form in cells but esterified into the *sn*-2 position of membrane glycerophospholipids, the participation of phospholipase A_2_ enzymes that liberate the fatty acid constitutes a limiting step for the synthesis of eicosanoids, a process which also depends on the expression levels and activity of the AA-metabolizing enzymes cyclooxygenases and lipoxygenases [2,3,4,5].

AA, which is usually the major polyunsaturated fatty acid in the membranes of innate immune cells, is not uniformly distributed among membrane glycerophospholipids. Rather, marked differences exist in the distribution of AA across several different phospholipid molecular species. Monocytes and macrophages exhibit a characteristic distribution of AA between phospholipid classes, with ethanolamine phospholipids (PE) constituting the richest AA-containing class, followed by choline glycerophospholipids (PC), and phosphatidylinositol (PI) [6,7,8]. Among particular molecular species within phospholipid classes, the ethanolamine plasmalogens are markedly enriched with AA [6,7,8,9,10,11]. This asymmetric distribution of AA in cells is also key for eicosanoid regulation because, depending on the original phospholipid source of the AA, certain eicosanoids may be produced in preference over others. For example, production of lipoxygenase metabolites in ionophore-activated human neutrophils [12] and zymosan-stimulated mouse peritoneal macrophages [8] appears to be strikingly associated with the mobilization of AA from PC, not PE or PI. By inference, these findings imply that not all AA-containing phospholipid pools may be accessible to the relevant phospholipase A_2_ affecting the AA release. Thus, depending on conditions, AA compartmentalization among different membrane pools may constitute a third limiting step for eicosanoid synthesis.

Incorporation of AA into cellular phospholipids does not primarily occur via de novo phospholipid synthesis but at a later step via fatty acid recycling of the *sn*-2 position of glycerophospholipids in the so-called Lands pathway [5,11,12,13,14,15]. In this pathway, the 2-lysophospholipids generated by constitutively active phospholipase A_2_ enzymes such as the calcium-independent group VIA enzyme (iPLA_2_β) [16,17,18,19,20] are utilized by AA-selective coenzyme A (CoA)-dependent acyltransferases to incorporate AA into phospholipids. Afterward, a further remodeling step, primarily involving direct transacylation reactions between phospholipids, serves to place the AA in the appropriate phospholipid pools [5,11,12,13,14]. These reactions proceed without energy expenses as no fatty acid activation by CoA is involved and are believed to be responsible for the asymmetric distribution of AA between phospholipid classes and molecular species and, especially, for the very high content of this fatty acid in ethanolamine plasmalogens [5,11,12,13,14].

CoA-independent transacylase (CoA-IT; EC 2.3.1.147) is a major enzyme involved in phospholipid AA remodeling in most cells. This enzyme catalyzes the CoA-independent transfer of AA and other polyunsaturates from, primarily, diacyl-PC to lyso-PE or lyso-PC [11,12,13,14]. CoA-IT manifests marked affinity for lysophospholipid acceptors containing an ether bond in the *sn*-1 position of the glycerol backbone, particularly ethanolamine lysoplasmalogens (1-alkenyl-2-lyso-glycerophosphoethanolamine) and alkyl-lyso-PC (1-alkyl-2-lysoglycerophospho-choline). This circumstance may explain why the AA content in ether-containing PC and PE species is generally higher than in their diacyl counterparts. Although the gene sequence of CoA-IT has not been identified so far, its activity has been relatively well-characterized in broken cell preparations, and pharmacological inhibitors have been identified [21,22,23]. The enzyme can also be followed in whole cells by determining the pattern of radiolabeling of PC versus PE. Like all phospholipase A_2_ enzymes, CoA-IT cleaves the *sn*-2 position of phospholipids, but it cannot be regarded as a bona fide phospholipase A_2_ enzyme because it does not produce a free fatty acid [13,14]. Given that some of the better known phospholipase A_2_s such as cytosolic group IVA phospholipase A_2_ (cPLA_2_α) or iPLA_2_β exhibit CoA-IT activity in in vitro assays [16], the proposal has been made that, in vivo, the CoA-IT reaction may represent an unidentified function of an otherwise described phospholipase A_2_ enzyme [14]. Based primarily on biochemical commonalities, i.e., membrane-bound, calcium-independent, group IVC cytosolic phospholipase A_2_ (cPLA_2_γ) has been suggested as a likely candidate [14]. cPLA_2_γ overexpression studies have provided in vivo evidence that the enzyme modulates phospholipid fatty acid composition, although it was not clarified whether the remodeling reactions involved were CoA-dependent or -independent [24].

In previous work from our laboratory, we used advanced mass spectrometry-based lipidomic approaches to study phospholipase A_2_-regulated phospholipid fatty acid metabolism (release and reincorporation mechanisms) in monocytes and macrophages responding to stimuli of the innate immune response [6,7,8,25,26,27,28,29]. In this work, we have employed similar approaches to unveil novel regulatory features of CoA-IT-mediated phospholipid remodeling responses. To characterize further the role of plasmalogen forms in these processes, we have taken advantage of the plasmalogen-deficient RAW cell variants generated by Zoeller and coworkers [30,31,32]. Our previous studies with these cells uncovered essential roles for ethanolamine plasmalogens in regulating phagocytosis [33] and in the execution of lipopolysaccharide (LPS)-primed responses [29].

In the current work, we show that overall phospholipid AA remodeling is essentially independent of the amount of plasmalogen present in the cells, suggesting that compartmentalization of AA in innate immune cells may depend primarily on the relative distribution of the fatty acid between classes (PE versus PC versus PI) rather than on specific molecular species within classes (i.e., alkenyacyl versus alkylacyl versus diacyl species). Moreover, our results also implicate cPLA_2_γ in regulating phospholipid AA remodeling.

## 2. Materials and Methods

### 2.1. Reagents

Cell culture medium was from Molecular Probes-Invitrogen (Carlsbad, CA, USA). Organic solvents (Optima^®^ LC/MS grade) were from Fisher Scientific (Madrid, Spain). Lipid standards were from Avanti (Alabaster, AL, USA) or Cayman (Ann Arbor, MI, USA). Silicagel G thin-layer chromatography plates were from Macherey-Nagel (Düren, Germany). [5,6,8,9,11,12,14,15-^3^H]Arachidonic acid (180 Ci/mmol) was from PerkinElmer (Boston, MA, USA). The group IVA phospholipase A_2_ (cytosolic phospholipase A_2_α; cPLA_2_α) inhibitor pyrrophenone [34] was synthesized and provided by Dr. Alfonso Pérez (Department of Organic Chemistry, University of Valladolid). The group VIA phospholipase A_2_ (calcium-independent phospholipase A_2_β; iPLA_2_β) inhibitors FKGK18 and GK436 and the secreted phospholipase A_2_ inhibitor GK241 were synthesized in the Kokotos laboratory [35,36,37]. All other reagents were from Sigma-Aldrich (Madrid, Spain).

### 2.2. Cell Culture

RAW264.7 macrophage-like cells and their ether phospholipid-deficient variants RAW.12 and RAW.108 (generously provided by Dr. R. A. Zoeller, Boston University) [30,31,32] and P388D_1_ macrophage-like cells (MAB clone, generously provided by Dr. E. A. Dennis, University of California at San Diego) [38,39,40] were grown in Dulbecco’s modified Eagle’s medium supplemented with 10% (v/v) fetal bovine serum, 100 U/mL penicillin, 100 μg/mL streptomycin, and 2 mM L-glutamine at 37 °C in a humidified atmosphere of 5% CO_2_, as previously described [41,42]. Mouse peritoneal macrophages from Swiss mice (University of Valladolid Animal House, 10–12 weeks old) were obtained by peritoneal lavage using 5 mL cold phosphate-buffered saline and cultured in RPMI 1640 medium with 10% fetal bovine serum, 100 U/mL penicillin, and 100 μg/mL streptomycin, as described elsewhere [43,44]. All procedures involving animals were undertaken under the supervision of the Institutional Committee of Animal Care and Usage of the University of Valladolid (No. 907046) and are in accordance with the guidelines established by the Spanish Ministry of Agriculture, Food, and Environment and by the European Union.

All experiments were conducted in serum-free media. When activated cells were used, the stimulus (150 µg/mL yeast-derived zymosan) was added 1 h after incubating the cells in serum-free media for the times indicated. Zymosan was prepared exactly as described [43,44]. Only zymosan batches that demonstrated no measurable endogenous phospholipase A_2_ activity, as measured by in vitro assay under different conditions [45,46], were used in this study. Cell protein content was quantified according to Bradford [47], using a commercial kit (BioRad Protein Assay). To generate classically (M1) or alternatively (M2) polarized macrophages, the cells were treated with LPS (250 ng/mL) plus interferon-γ (500 U/mL) or with interleukin-4 (20 ng/mL) plus interleukin-13 (20 ng/mL) for 8 h, respectively [48]. When required, radiolabeling of the cells with [^3^H]AA was achieved by including 0.25 µCi/mL [^3^H]AA during the overnight adherence period (20 h). Labeled AA that had not been incorporated into cellular lipids was removed by washing the cells four times with serum-free medium containing 0.5 mg/mL albumin. The determination of [^3^H]AA release from activated cells was carried out exactly as described [41,42,49].

### 2.3. Quantitative PCR

Total RNA was extracted from the cells with TRIzol reagent (Invitrogen) according to the manufacturer’s instructions, and 2 µg RNA was reverse transcribed using random primers and oligo d(T) and Moloney murine leukemia virus reverse transcriptase (Ambion, Austin, TX, USA). Quantitative PCR was carried out with an ABI 7500 machine (Applied Biosystems, Carlsbad, CA, USA) using Brilliant III Ultra-Fast SYBR Green qPCR Master Mix (Agilent Technologies, Santa Clara, CA, USA). Cycling conditions were as follows: 1 cycle at 95 °C for 3 min and 40 cycles at 95 °C for 12 s, 60 °C for 15 s, and 72 °C for 28 s. The replicates were averaged, and fold induction was determined in ΔΔCt-based fold-change calculations, with cyclophilin A as a control [50]. Primer sequences are available upon request.

### 2.4. Small Interfering RNA (siRNA) Transfection

The procedure for transient transfection of RAW264.7 cells with siRNAs targeting group IVC phospholipase A_2_ (cytosolic phospholipase A_2_γ; cPLA_2_γ) was adapted from Valdearcos et al. [51]. Briefly, 3 × 10^5^ cells were transfected with siRNAs (20 nM) (sequence: 5′-(GGAGGAGAGAGAGGAAGAGAA)TT-3′; Eurofins Genomics, Ebersberg, Germany) in the presence of 5 µl/mL Lipofectamine^TM^ RNAiMAX (Invitrogen) under serum-free conditions for 5 h. Afterward, 5% serum was added and the cells were maintained at normal culture conditions for 24 h. A scrambled siRNA was used as a negative control (sequence: 5′-UGGUUUACAUGUCGACUAA-3′).

### 2.5. Gas Chromatography/Mass Spectrometry (GC/MS) Analyses

Total lipids from approximately 10^7^ cells were extracted according to Bligh and Dyer [52]. After addition of internal standards, phospholipids were separated from neutral lipids by thin-layer chromatography, using *n*-hexane/diethyl ether/acetic acid (70:30:1, v/v/v) [53]. The phospholipid classes (PC, choline-containing phospholipids; PE, ethanolamine-containing phospholipids; PI, phosphatidylinositol; and PS, phosphatidylserine) were separated twice with chloroform/methanol/28% (w/w) ammonium hydroxide (60:37.5:4, v/v/v) as the mobile phase [54]. Fatty acid methyl esters were obtained from the various lipid fractions by transmethylation with 0.5 M KOH in methanol for 60 min at 37 °C [55,56,57,58,59]. Analysis was carried out using an Agilent 7890A gas chromatograph coupled to an Agilent 5975C mass-selective detector operated in electron impact mode (EI, 70 eV), equipped with an Agilent 7693 autosampler and an Agilent DB23 column (60 m length × 0.25 mm internal diameter × 0.15 µm film thickness). Data analysis was carried out with the Agilent G1701EA MSD Productivity Chemstation software, revision E.02.00 [55,56,57,58,59] (Agilent Technologies, Santa Clara, CA, USA).

### 2.6. Liquid Chromatography/Mass Spectrometry (LC/MS) Analyses of Phospholipids

Lipids were extracted according to Bligh and Dyer [52], and internal standards were added. The samples were redissolved in 50 μL of hexanes/2-propanol/water (42:56:2, v/v/v), and 40 μL was injected into an Agilent 1260 Infinity high-performance liquid chromatograph equipped with an Agilent G1311C quaternary pump and an Agilent G1329B autosampler (Agilent Technologies, Santa Clara, CA, USA). The column was a FORTIS HILIC (150 × 3 mm, 3 μm particle size) (Fortis Technologies, Geston, UK), protected with a Supelguard LC-Si (20 mm × 2.1mm) cartridge (Sigma-Aldrich, Madrid, Spain). The mobile phase consisted of a gradient of solvent A (hexanes/2-propanol, 30:40, v/v) and solvent B (hexanes/2-propanol/20 mM ammonium acetate in water, 30:40:7, v/v/v). The gradient started at 75% A from 0 to 5 min, then decreased from 75% A to 40% A at 15 min and from 40% A to 5% A at 20 min, was held at 5% until 40 min, and increased to 75% at 41 min. The column was then re-equilibrated by holding at 75% A for an additional 14 min before the next sample injection. The flow rate through the column was fixed at 400 μL/min, and this flow entered into the electrospray ionization interface of an AB/Sciex QTRAP 4500 hybrid triple quadropole mass spectrometer operated in negative ion mode (Applied Biosystems, Carlsbad, CA, USA). Source parameters were as follows: ion spray voltage, −4500 V; curtain gas, 30 psi; nebulizer gas, 50 psi; desolvation gas, 60 psi; and temperature, 425 °C. Phospholipid species were detected as [M–H]^−^ ions except for choline phospholipids, which were detected as [M+CH_3_COO^−^]^−^ adducts and were identified by comparison with previously published data [6,7,8,25,26,27,28,29].

### 2.7. Liquid Chromatography/Mass Spectrometry (LC/MS) Analyses of Eicosanoids

Analysis of eicosanoids by LC/MS was carried out exactly as described elsewhere [8,27], using an Agilent 1260 Infinity high-performance liquid chromatograph equipped with an Agilent G1311C quaternary pump and an Agilent G1329B Autosampler, coupled to an API2000 triple quadrupole mass spectrometer (Applied Biosystems, Carlsbad, CA, USA). Quantification was carried out by integrating the chromatographic peaks of each species and by comparing with an external calibration curve made with analytical standards [8,27].

### 2.8. Measurement of Phospholipid Arachidonate Remodeling

**Scheme 1:**
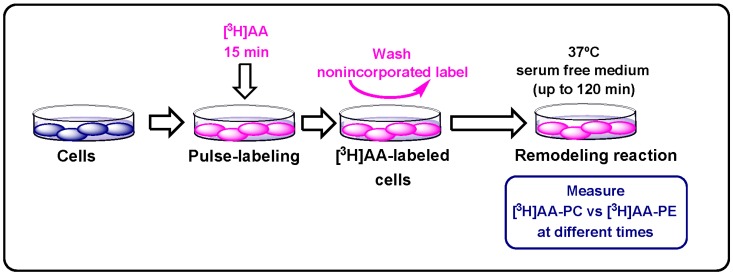
Measurement of arachidonic acid (AA) movement from choline glycerophospholipids (PC) to ethanolamine phospholipids (PE).

This was carried out exactly as described by Pérez et al. [60]. Briefly, the cells were pulse labeled with 1 nM [^3^H]AA (0.25 µCi/mL) for 15 min at 37 °C. The cells were then washed with medium containing 0.5 mg/mL bovine serum albumin to remove the non-incorporated label. Afterward, the cells were placed in serum-free medium and incubated at 37 °C for the indicated periods of time. After lipid extraction, phospholipid classes were separated by thin-layer chromatography as indicated above. The spots corresponding to each phospholipid class were cut out and assayed for radioactivity by liquid scintillation counting. Scheme 1 provides a graphical description of this kind of experiment.

### 2.9. Statistical Analysis

All experiments were carried out at least three times with incubations in duplicate or triplicate, and the data are expressed as means ± SEM. Statistical analysis was carried out by Student’s *t*-test, with *p* < 0.05 taken as statistically significant.

## 3. Results

### 3.1. AA Distribution in RAW264.7 Cells and Plasmalogen-Deficient Variants

**Figure 1:**
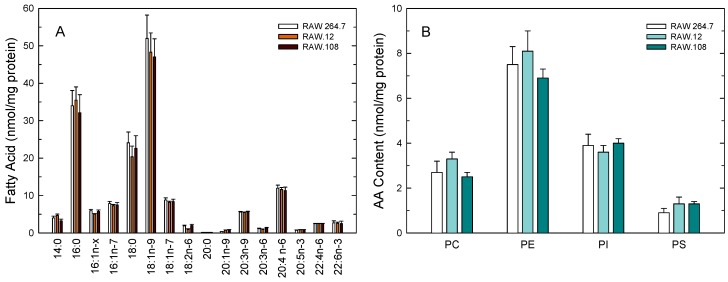
Phospholipid fatty acid composition of RAW 264.7 cells and plasmalogen-deficient variants: (**A**) The profiles of major fatty acids in RAW 264.7 cells (open bars), RAW.12 (light brown), and RAW.108 (dark brown) were determined in the phospholipid fraction by GC-MS after converting the fatty acid glyceryl esters into fatty acid methyl esters. 16:1*n*-x denotes a mix of the *n*-9 and *n*-10 isomers, which elute together. (**B**) The phospholipid classes of RAW 264.7 cells (open bars), RAW.12 (light turquoise), and RAW.108 (dark turquoise) were separated, and their AA content was determined by GC/MS. Results are shown as means ± S.E.M. (n = 3).

Figure 1A compares the phospholipid fatty acid composition of RAW264.7 cells and the plasmalogen-deficient variants RAW.12 and RAW.108, as assessed by GC/MS. Fatty acids are designated by their number of carbon atoms, and their number of double bonds are designated after a colon. To differentiate isomers, the n−x (n minus x) nomenclature is used, where n is the number of carbons of a given fatty acid and x is an integer which, subtracted from n, gives the position of the last double bond of the molecule. The AA content was very similar in all three cell types tested. Also, no significant variations were detected in any other fatty acid, including the polyunsaturates of the *n*–3 series. When the various phospholipid classes were separated and analyzed for AA content, PE was found to constitute the major AA-containing class in all three types, representing almost half of total AA present in these cells. The fatty acid was found at comparable levels in PI and PC, and lower amounts were found in PS. No other phospholipid class contained significant amounts of AA (Figure 1B). The relative distribution of AA among phospholipid classes of RAW264.7 cells and its plasmalogen-deficient variants, with PE predominating, is consistent with previous data for other phagocytic cells such as human monocytes [6] and murine peritoneal macrophages [7,8]. Collectively, these results show that cellular plasmalogen content influences neither cellular AA levels nor the relative distribution of the fatty acid among phospholipid classes.

**Figure 2:**
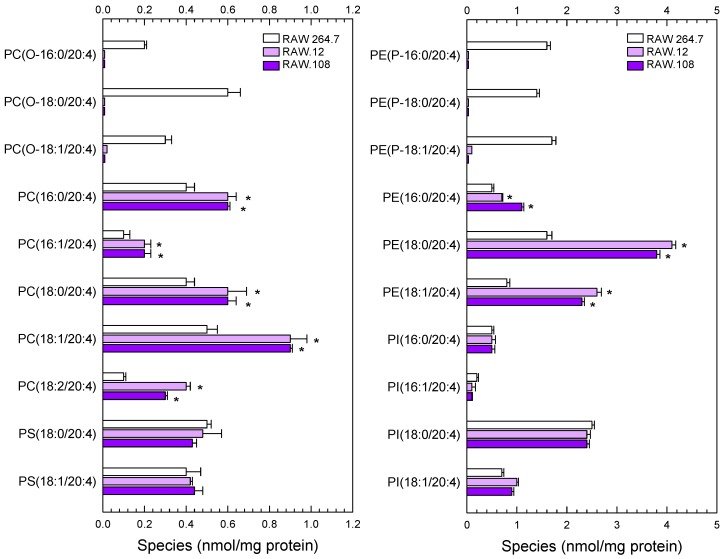
AA-containing phospholipid species in RAW 264.7 cells and plasmalogen-deficient variants: The profile of AA-containing PC, PS (left panel), PE, PI (right panel) was determined for RAW 264.7 cells (open bars), RAW.12 cells (light purple bars), and RAW.108 cells (dark purple bars) by LC/MS. Results are shown as means ± S.E.M. (n = 3). * *p* < 0.05, significantly different from the corresponding species of RAW264.7 cells.

The distribution of AA between phospholipid molecular species was measured by LC/MS (Figure 2). Fatty chains within phospholipids are designated by their number of carbon atoms, and their number of double bonds are designated after a colon. A designation of O- before the first fatty chain indicates that the *sn*-1 position is ether-linked, whereas a P- designation indicates a plasmalogen form (*sn*-1 vinyl ether linkage) [61]. The results showed that PE plasmalogens constituted the major reservoir of this fatty acid in RAW 264.7 cells (Figure 2). High amounts of AA were also found in one particular PI species, namely PI(18:0/20:4), and in diacyl-PE species. Lower AA amounts were found in several PC and PS species (Figure 2). Interestingly, despite the plasmalogen deficiency of RAW.12 and RAW.108 cells, the AA distribution by the phospholipid class in these cells was preserved due to a compensatory elevation of AA in diacyl species of PE and PC compared to wild type RAW 264.7 cells (Figure 2).

### 3.2. Importance of Plasmalogen Content for Phosphospholipid AA Remodeling

**Figure 3:**
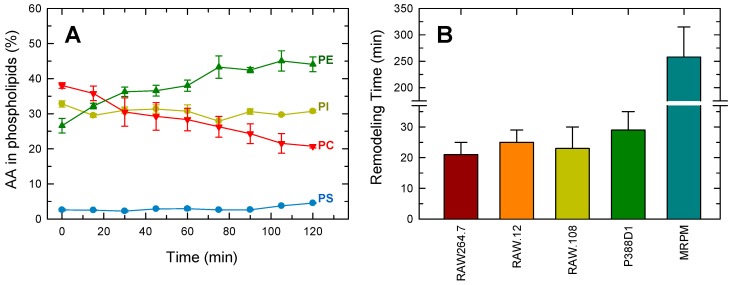
Phospholipid AA remodeling in RAW 264.7 cells and plasmalogen-deficient variants: (**A**) RAW 264.7 cells were pulse-labeled with [^3^H]AA, washed, and incubated without label for the indicated periods of time. Phospholipids were separated into classes by thin-layer chromatography. The radioactivity incorporated into each phospholipid class was determined by scintillation counting and is given as a percentage of the radioactivity present in phospholipids. (**B**) AA remodeling was analyzed for different cell types, and the remodeling time (time at which the radioactivity content of PC equals that of PE) was determined. Results are shown as means ± S.E.M. (n = 6 for panel A; n = 3 for panel B). MRPM, mouse resident peritoneal macrophages.

In mammalian cells, plasmalogen enrichment with AA is thought to occur primarily via CoA-IT-mediated reactions, which transfer a fatty acyl moiety from a phospholipid donor, primarily AA-containing PC species, to a lysophospholipid acceptor, very often an ethanolamine lysoplasmalogen, without using CoA or forming a free fatty acid intermediate [11,12,13,14]. This reaction also appears to be instrumental for AA mobilization responses, as inhibition of CoA-IT leads to marked inhibition of AA release [8,23,62]. To characterize this route, RAW 264.7 cells were labeled with [^3^H]AA for 15 min and, after extensive washing to remove non-incorporated fatty acid, the movement of labels between phospholipid classes was analyzed. Immediately after the 15-min labeling period, PC was the major [^3^H]AA-containing phospholipid, followed by PI and PE. [^3^H]AA incorporation into PS was considerably lower (Figure 3A). The amount of labeled AA in PC underwent a rapid decrease with time, which was paralleled by an increase of similar magnitude of AA in PE, reflecting the action of CoA-IT. Levels of labeled AA in PI and PS remained unchanged along the time course of the experiment. To make direct comparisons between various conditions and in accord with previous work [55] we have defined the time at which the amount of [^3^H]AA in PC equals that in PE as the “remodeling time” and found it to be 21 ± 4 min (mean ± S.E.M., n = 6). Importantly, examination of the rate of AA remodeling from PC to PE in the plasmalogen-deficient variants RAW.12 and RAW.108 revealed essentially the same kinetics as in RAW 264.7 cells and, hence, nearly identical remodeling times (Figure 3B). Thus, these results indicate that phospholipid AA remodeling from PC to PE is not influenced by the cellular plasmalogen content. For comparative purposes, remodeling experiments under identical conditions were also carried out using another murine macrophage-like cell line, P388D_1_, and using resident murine peritoneal macrophages. In keeping with previous estimates [63,64,65], the remodeling time of P388D_1_ cells was found to be similar to that of RAW 264.7 cells and their variants and considerably lower than that of murine peritoneal macrophages (Figure 3B).

### 3.3. Role of Plasmalogens in Functional Responses of Macrophages to Receptor Stimulation

**Figure 4:**
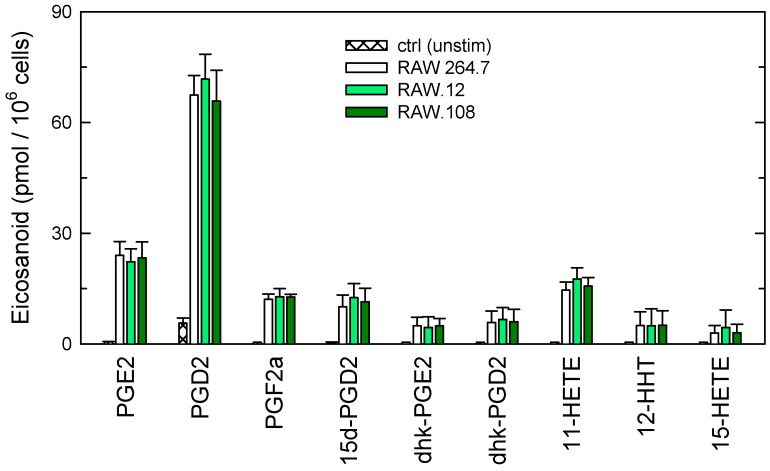
Eicosanoid production by RAW 264.7 cells and plasmalogen-deficient variants: RAW 264.7 cells (open bars), RAW.12 cells (light green bars), and RAW.108 cells (dark green bars) were stimulated with 150 µg/mL zymosan for 8 h. Afterward, extracellular media was removed and analyzed for eicosanoid levels by LC/MS. Eicosanoids produced by untreated RAW 264.7 cells (crosshatched bars) are shown for comparison. Results are shown as means ± S.E.M. (n = 3). PGE2, prostaglandin E_2_; PGD2, prostaglandin D_2_; PGF2a, prostaglandin F_2α_; 15d-PGD2, 15-deoxy-Δ^12,14^-prostaglandin D_2_; dhk-PGE2, 13,14-dihydro-15-keto-prostaglandin E_2_; dhk-PGD2, 13,14-dihydro-15-keto- prostaglandin D_2_; 11-HETE, 11-hydroxyeicosatetraenoic acid; 12-HHT, 12-hydroxyheptadecatrienoic acid; 15-HETE, 15-hydroxyeicosatetraenoic acid.

In previous work from our laboratory, we took advantage of the RAW 264.7 cells and the plasmalogen-deficient variants RAW.12 and RAW.108 to establish the key roles of ethanolamine plasmalogens in regulating the phagocytic activity [33] and in the execution of LPS-primed responses [29] of macrophages. Here, we extended our studies on the role of plasmalogens in cellular physiology by studying other functional responses of macrophages. Figure 4 shows the quantitative analysis of eicosanoids produced by zymosan-stimulated cells. The profile of eicosanoids produced was similar, both qualitatively and quantitatively, to that previously reported by Buczynski et al. [66]. Only products of the cyclooxygenase pathway were found, with PGD_2_ constituting the major eicosanoid detected. Eicosanoid production was the same in the RAW 264.7 cells as in the plasmalogen-deficient variants (Figure 4), thus suggesting that plasmalogen status does not influence the eicosanoid biosynthetic response of the macrophages.

**Figure 5:**
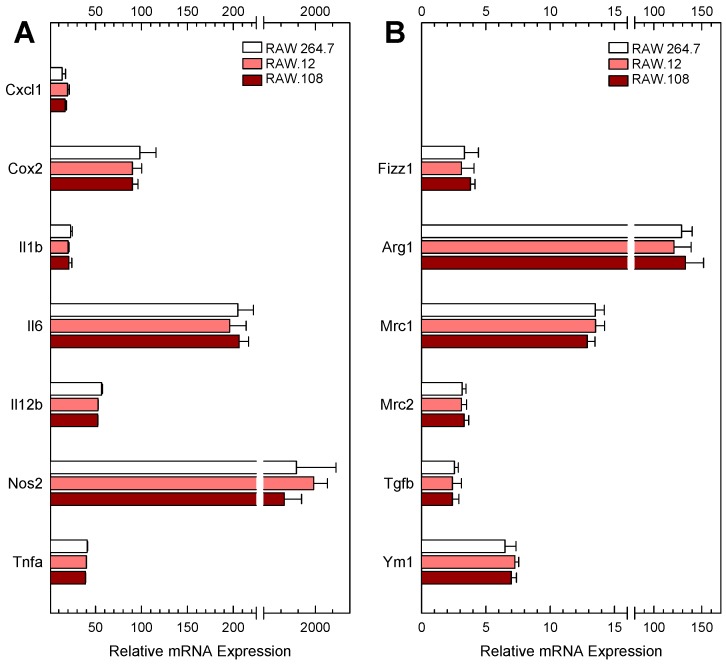
Macrophage polarization through the classic pro-inflammatory (M1) and alternative (M2) pathways: RAW 264.7 cells (open bars) and their plasmalogen-defective variants RAW.12 (light red bars) and RAW.108 (dark red bars) were treated for 8 h with LPS (250 ng/mL) plus interferon-γ (500 U/mL) (**A**) or with interleukin-4 (20 ng/mL) plus interleukin-13 (20 ng/mL) (**B**) to generate classically (M1) or alternatively (M2) polarized macrophages, respectively. Afterward, the expression of the indicated markers was studied by qPCR. Data are relative to basal expression levels and normalized to cyclophilin A. A representative experiment is shown, and the data are expressed as means ± S.E.M. of three individual replicates.

In the next series of experiments, we treated the cells with LPS/interferon-γ or interleukin-4/interleukin-13 to induce polarization/activation of the macrophages to pro-inflammatory (M1) or anti-inflammatory (M2) phenotypes [48], respectively, and changes in the gene expression levels of various markers associated to each phenotype were assessed by qPCR. Figure 5 shows that there were no differences between RAW 264.7 cells and the plasmalogen-deficient variants in the expression levels of any of the genes assayed.

These results, along with our previous data [29,33], underscore the differential involvement of plasmalogens in some, but not all, responses of macrophages, thus reflecting some sort of biological specificity of this kind of phospholipids.

### 3.4. Studies Utilizing AA-enriched Cells

**Figure 6:**
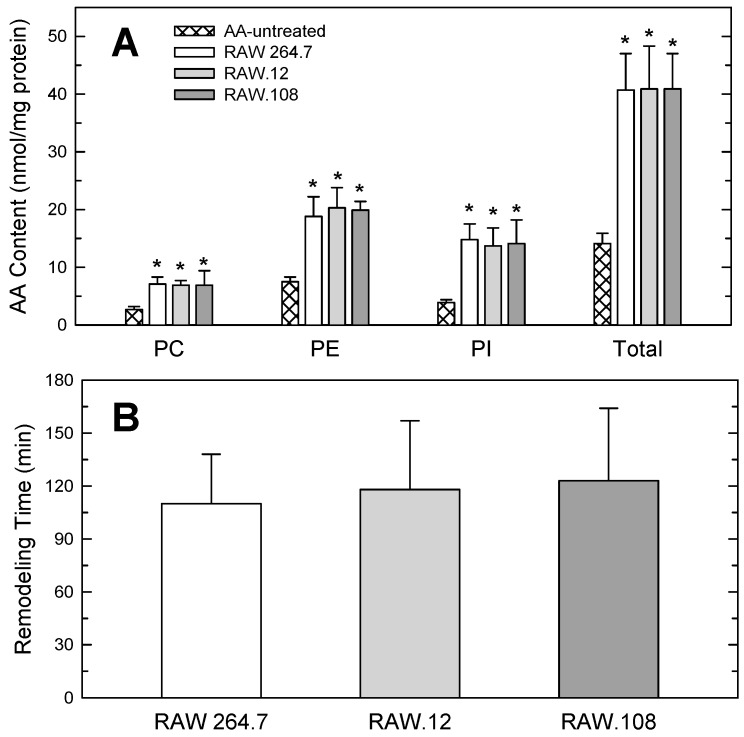
Phospholipid AA remodeling in AA-enriched cells: (**A**) RAW 264.7 cells (open bars), RAW.12 cells (light grey bars), and RAW.108 cells (dark grey bars) were incubated in media containing 25 µM exogenous AA for 48 h. Afterward, cellular AA content in phospholipids was analyzed by GC/MS. The AA content of otherwise untreated RAW 264.7 cells (cross-hatched bars) is shown for comparison. (**B**) AA remodeling was analyzed in the AA-enriched cells, and the remodeling time (time at which the radioactivity content of PC equals that of PE) was determined. Results are shown as means ± S.E.M. (n = 3). * *p* < 0.05, significantly different from AA-untreated cells.

As a consequence of continuous growth in culture, cell lines are known to exhibit diminished levels of polyunsaturated fatty acids, including AA, compared to normal cells [67,68]. For example, RAW 264.7 cells contain 2–3-fold less AA than murine resident peritoneal macrophages [58]. Since CoA-IT-driven fatty acid remodeling reactions determine the distribution of AA among membrane phospholipids, we reasoned that the much faster remodeling observed in RAW 264.7 cells compared to normal macrophages could be related to their relative deficiency in AA. To test this possibility, RAW 264.7 cells and the plasmalogen-deficient variants were cultured in media supplemented with 25 µM AA (complexed with bovine serum albumin at a 2:1 ratio) for 48 h. This procedure resulted in the cells increasing their AA content by about 2–3-fold, thus reaching levels comparable to those found in normal peritoneal macrophages [58]. Figure 6A compares the distribution of AA between phospholipid classes in the AA-enriched cells. Importantly, all of the AA-containing phospholipid classes in wild type RAW 264.7 cells and their variants incorporated exogenous AA to a similar extent. This resulted in the relative distribution profile of the fatty acid by class being preserved, i.e., PE comprising approximately 50% of cellular AA and PI constituting the second richest AA phospholipid, followed closely by PC. After the AA-enrichment period, the cells were spiked with [^3^H]AA and the movement of AA from PC to PE was followed at different times. Notably, under these conditions, the remodeling process was markedly slowed in both wild type and plasmalogen-deficient variants, with a remodeling time of approx. 2 h, i.e., comparable to that of resident peritoneal macrophages (*cf*. Figure 3B and Figure 6B). These data suggest that endogenous cellular levels of AA, not plasmalogen status, determines the rate of remodeling of AA among phospholipids.

A key step in phospholipid AA remodeling is the continuous generation of lysophospholipid moieties by phospholipase A_2_ enzymes, which act as fatty acid acceptors for the subsequent CoA-IT-mediated transacylation reaction. Since macrophage activation by receptor-directed agonists, including zymosan, results in noticeable elevations of lysophospholipid levels compared to unstimulated cells [8,27,44,69,70], we investigated whether the activation state of the cells, with its attendant elevation of cellular lysophospholipid levels, exerted any influence on their phospholipid AA remodeling rate. To this end, the cells were labeled with [^3^H]AA for 15 min and, after extensive washing, they were untreated (control incubations) or treated with zymosan (150 µg/mL) for different time periods and the transfer of label from PC to PE was measured. The experiments were carried out in otherwise untreated cells and in exogenous AA-treated cells, and both wild type and plasmalogen variants were used. No effect of cell activation could be ascertained on phospholipid AA remodeling under any of these conditions. The remodeling time of zymosan-activated cells was the same as that of resting cells, either using wild-type cells, the plasmalogen-deficient variants, or cells preloaded with exogenous AA. Thus, phospholipid AA remodeling rate is independent of the activation state of the cells.

### 3.5. Phospholipase A_2_ Inhibition Studies

Earlier studies attempting to identify the origin of the lysophospholipid acceptors used in the CoA-IT-mediated transacylation reaction took advantage of PLA_2_ inhibitors available at that time; however, given the uncertain specificity of some of the inhibitors used, these studies did not provide unambiguous responses [71,72]. Recently, much more selective phospholipase A_2_ inhibitors with improved properties have been developed [73] and we have used these to re-evaluate the involvement of phospholipase A_2_ isoforms. The inhibitors used were pyrrophenone for cPLA_2_α (at least 3 orders of magnitude more potent for cPLA_2_α than for iPLA_2_β or sPLA_2_ enzymes) [34]; FKGK18 for iPLA_2_β (at least 200 and 400 times more potent for iPLA_2_β than for cPLA_2_α and sPLA_2_s, respectively) [35]; GK436, also for iPLA_2_β (at least 1000-fold more potent for iPLA_2_β than for cPLA_2_α, and no appreciable effect on sPLA_2_ enzymes) [36]; and GK241 for sPLA_2_ (inhibits IIA and V forms, lacking appreciable inhibition against cPLA_2_α, iPLA_2_β, or any other sPLA_2_ form) [37]. However, neither of these inhibitors had any measurable effect on the remodeling time of RAW 264.7 cells, thus providing further additional support for the lack of involvement of cPLA_2_α, iPLA_2_β, or sPLA_2_-IIA/V in phospholipid AA remodeling.

**Figure 7:**
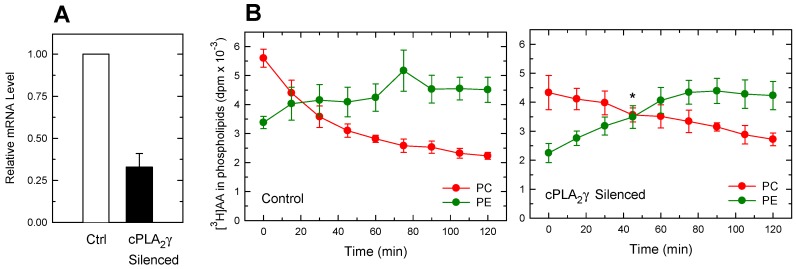
cPLA_2_γ role in phospholipid AA remodeling: (**A**) PCR analysis of cPLA_2_γ expression in RAW 264.7 cells. (**B**) Phospholipid AA remodeling was analyzed in cells treated with a scrambled siRNA (Control) or siRNA targeting cPLA_2_γ (cPLA_2_γ Silenced). Results are shown as means ± S.E.M. (n = 5). The asterisk in the Figure 7B right panel denotes that the remodeling time of cPLA_2_γ-silenced cells is significantly different from that of control cells (* *p* < 0.05).

**Figure 8:**
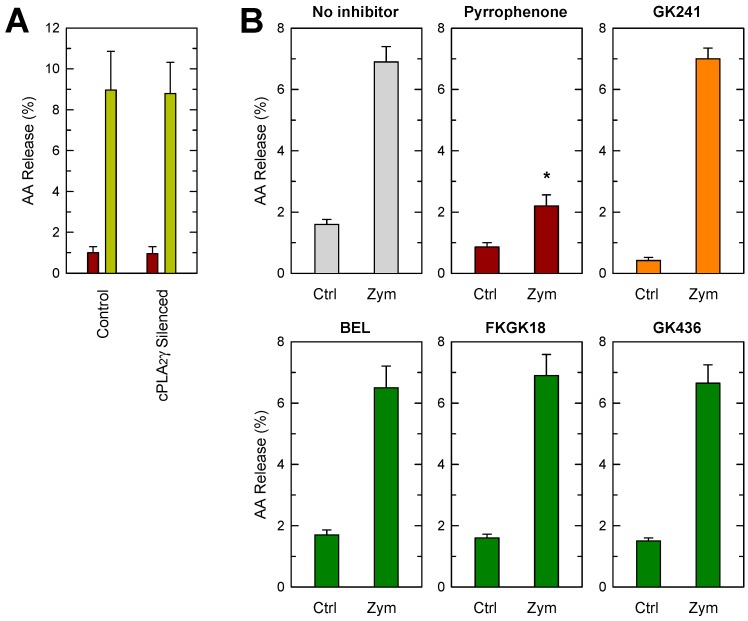
Stimulated AA mobilization in RAW 264.7 cells: (**A**) [^3^H]AA-labeled cells, treated with a scrambled siRNA (Control) or siRNA for cPLA_2_γ (cPLA_2_γ Silenced), were left untreated (dark red bars) or treated with 150 µg/mL zymosan (dark yellow bars) for 1 h. (**B**) The cells, labeled with [^3^H]AA, were preincubated with either 2 µM pyrrophenone, 10 µM GK241, 5 µM bromoenol lactone (BEL), 10 µM FKGK18, 5 µM GK436, or neither (no inhibitor) for 30 min. Afterward they were untreated (Ctrl) or treated with 150 µg/mL zymosan for 1 h. Results are shown as means ± S.E.M. (n = 3). * *p* < 0.05, significantly different from zymosan-stimulated cells in the absence of inhibitors.

Recently, it has been speculated that a lesser known member of the group IV phospholipase A_2_ family of enzymes, i.e., the group IVC form, also known as cytosolic phospholipase A_2_γ (cPLA_2_γ), could be involved in phospholipid AA remodeling [14]. This suggestion is made primarily on the basis of the biochemical properties of cPLA_2_γ (i.e., Ca^2+^-independent, manifests measurable CoA-independent transacylation activity in vitro, and permanently associated with membranes) [14]. To begin to address this question, conditions were established to achieve silencing of cPLA_2_γ by siRNA technology in RAW 264.7 cells. Since we have been unable to find reliable antibodies against murine cPLA_2_γ, the efficiency of siRNA knockdown was judged by qPCR. Using this technique, we were able to achieve as much as a 70–75% decrease in cPLA_2_γ mRNA under our conditions (Figure 7A). Strikingly, even with this incomplete cPLA_2_γ silencing, cells still exhibited clear defects in AA remodeling from PC to PE, reflected by a statistically significant increase of their remodeling time (Figure 7B). Thus, these data provide strong evidence for the involvement of cPLA_2_γ in phospholipid AA remodeling in macrophages. Conversely, upon stimulation with zymosan, cells deficient in cPLA_2_γ released similar amounts of AA as control cells (Figure 8A), thus indicating that the enzyme is not a significant regulator of the AA release response. In keeping with previous data [8,41,42,74,75,76,77], AA-release experiments conducted in parallel in the presence of the phospholipase A_2_ inhibitors described above confirmed the role of cPLA_2_α but not of sPLA_2_ or iPLA_2_β in AA release, as only inhibition of cPLA_2_α resulted in blockade of the response (Figure 8B). In these experiments, we also used bromoenol lactone (BEL) for comparative purposes with data in bibliography [32,74]. BEL is a widely used irreversible inhibitor of calcium-independent phospholipase A_2_s, with little or no effect on calcium-dependent enzymes [78,79], although with potential off-target effects depending on cell type [80,81]. In our hands, BEL did not have any effect on zymosan-stimulated AA release (Figure 8B). Collectively, these results provide evidence for separate roles for the group IV cytosolic family members cPLA_2_α and cPLA_2_γ in cellular AA homeostasis; while the former regulates AA release but not phospholipid AA remodeling, the latter does the opposite.

## 4. Discussion

Phospholipid AA remodeling is necessary for cells of the innate immune system to distribute fatty acid within the appropriate cellular pools for its subsequent mobilization by phospholipase A_2_ enzymes. This is a key aspect in eicosanoid regulation because the nature and amount of eicosanoids produced under activation conditions may ultimately depend on the composition and subcellular localization of the phospholipid pool where the AA-hydrolyzing phospholipase A_2_ acts [1,2]. In innate immune cells, ether phospholipids, particularly the ethanolamine plasmalogens, are strikingly enriched in AA, which is thought to be due to the fact that these phospholipids are major acceptors for fatty acid transfer reactions between phospholipids that help shape the final distribution of the fatty acid in cells [11,12,13,14]. While the enrichment of ether phospholipids with AA suggests a key role for this kind of phospholipids in AA homeostasis, their role still remains relatively obscure. In fact, receptor stimulation of AA mobilization in plasmalogen-deficient cells is similar to that of normal cells [29,32], and none of the major AA-releasing phospholipase A_2_s described to date have been found to exhibit any particular preference for substrates containing an *sn*-1 ether bond [16]. Although these data suggested that plasmalogens may not be essential for the phospholipase A_2_-mediated AA mobilization process itself, the possibility remained that they are still instrumental for placing the fatty acid in the appropriate subcellular localizations via phospholipid transacylation reactions. Unexpectedly, the results presented in this study show that this is also not the case. We find no difference between plasmalogen-deficient and otherwise normal cells, thus suggesting that plasmalogen status has no influence on phospholipid AA remodeling. It should be noted in this regard that the relative distribution of AA among phospholipid classes (i.e., PE versus PC versus PI) is maintained in the plasmalogen-deficient cells because, in these cells, there is a compensatory elevation of AA levels in diacyl species. This is an important concept because it suggests that, in terms of overall AA distribution, it is the substituent at the *sn*-3 position of the phospholipid (i.e., ethanolamine versus choline versus inositol versus serine) and not the chemical nature of the *sn*-1 bond (acyl versus alkyl versus alkenyl) that determines the incorporation of AA. Consistent with this notion, AA transacylation reactions involve only ethanolamine- and choline-containing phospholipids, thus indicating some sort of specificity at the level of the *sn*-3 substituent.

While the lack of *sn*-1 influence on the AA transacylation reaction in cells is unanticipated, it should be noted that CoA-IT, the enzyme catalyzing these transacylation reactions, may use 1-acyl-PE just as well as 1-alkenyl-PE [11,12,13,14] and this, in fact, may allow to explain satisfactorily why the plasmalogen-deficient variants are able to compensate their deficiency by directing the AA to diacyl phospholipids. On the other hand, the finding that plasmalogen-deficient cells and normal cells mobilize AA similarly in spite of their composition being so different at the molecular species level provides strong support to the idea that cells can sustain critical reactions with many different lipid compositions rather than a single composition [82]. Furthermore, our results also raise the intriguing possibility that the enrichment of plasmalogens with AA might not be necessarily related to regulatory aspects of AA homeostasis and eicosanoid metabolism but to biophysical effects and interaction of the phospholipid with other membrane components to sustain different biological responses. Our collective findings showing that plasmalogens participate in the execution of some responses [29,33] but not in others (this study) are fully consistent with this view. AA-containing ethanolamine plasmalogens are frequently found as components of specific membrane microdomains called lipid rafts [83,84,85]. The relative plasmalogen content within these domains may affect key properties such as fluidity, tendency to fusion, packing, thickness, and density, thereby influencing the biological behavior of membrane rafts in membrane transport and transmembrane signaling [83,84,85].

CoA-IT catalyzes the enzymatic step that is unique to the phospholipid AA remodeling pathway, i.e., the direct transfer of AA from a phospholipid donor to a lysophospholipid acceptor in the absence of CoA or ATP [11,12,13,14]. CoA-IT has been defined as a membrane-bound, calcium-independent enzyme. Based on biochemical and mechanistic commonalities, Yamashita and coworkers suggested that CoA-independent transacylation reactions in cells are catalyzed by (an) enzyme(s) of the phospholipase A_2_ family and speculated that group IVC cytosolic phospholipase A_2_ (also called cytosolic phospholipase A_2_γ, cPLA_2_γ) is a possible candidate [14,86,87]. The same authors noted that the cPLA_2_γ-catalyzed transacylase reaction works better when the AA donor is lysoPC instead of AA-containing diacyl-PC. On the other hand, Stewart et al. [88] noted that the enzyme has higher lysophospholipase activity than phospholipase A_2_ activity. However, conditions of in vitro specificity assays are not necessarily translatable to the in vivo situation, where compartmentalization of substrates and products and the presence of competing enzymes may dramatically modify the specificities reported.

By using siRNA technology to specifically knock down cellular expression levels of cPLA_2_γ, we have tested experimentally the proposed involvement of cPLA_2_γ in regulating phospholipid AA remodeling. Our results clearly indicate that this is the case, as cells which were made deficient in cPLA_2_γ transfer AA from PC to PE significantly more slowly than control cells. These results constitute, to the best of our knowledge, the first report attributing cellular AA remodeling activity to a well-characterized molecular entity, i.e., the cPLA_2_γ enzyme. Thus, the finding is significant because it makes now possible to apply molecular biology approaches, such as overexpression or deletion, which should significantly expand our knowledge about the cellular and molecular regulation of phospholipid AA remodeling reactions. At this point, we cannot indicate whether cPLA_2_γ participates in the CoA-independent transacylation reaction by providing the lyso acceptors that initiate the reaction, by directly catalyzing the fatty acid transacylation, or by acting at both levels. Clearly, further work should be conducted to clarify these mechanistic issues and whether the enzyme is constitutively active or its activity increases after agonist stimulation [24]. Also, since our data clearly show that selective inhibition of cPLA_2_γ by siRNA slows but does not eliminate phospholipid AA remodeling, it seems likely that other enzymes in addition to cPLA_2_γ operate as CoA-IT in cells, and further work should be undertaken to identify them.

Another striking feature of this work is the finding that the rate of AA remodeling from PC to PE appears to be independent of the state of activation of the cells. We could not observe any change in the rate of AA remodeling from PC to PE in zymosan-stimulated cells compared to unstimulated cells. This finding was somehow unexpected because lysophospholipid availability initiates phospholipid AA remodeling in cells and zymosan-activated macrophages increase their intracellular lysophospholipid content as a consequence of cPLA_2_α activation [8,27,29]. Lack of an effect of cell stimulation on CoA-IT-mediated phospholipid remodeling is at variance with previous results in phorbol ester-stimulated platelets [89], tumor necrosis factor α-stimulated neutrophils [90], and antigen-stimulated mast cells [91] but is consistent with work in neutrophils on the regulation of platelet-activating factor synthesis via transacylation reactions, where it was found that CoA-IT activity did not increase as a consequence of cell activation but that regulation occurred at the level of increased substrate availability [92]. In this regard, we also report in this paper that increasing the cellular levels of AA results in decreased rates of CoA-mediated phospholipid AA remodeling. It is thus tempting to speculate with a scenario wherein an AA-containing phospholipid that is present in the AA-enriched cells but not in the otherwise normal cells may act to regulate CoA-IT-mediated transacylation reactions by directly impinging on the enzyme and/or by regulating substrate availability. We have previously shown that a short-lived AA-containing phospholipid produced by activated cells, namely 1,2-diarachidonoyl-glycerophosphoinositol, is able to regulate macrophage responses to innate immune stimuli [7].

## 5. Conclusions

We demonstrate in this work that, although plasmalogens constitute the major reservoirs of AA in normal cells, CoA-IT-mediated phospholipid AA remodeling reactions responsible for the asymmetric distribution of the fatty acid in the various phospholipid pools are not influenced by the plasmalogen content of cells. Compartmentalization of AA in cells appears to depend primarily on headgroup composition of membrane phospholipids rather than on specific molecular species. In addition, our work implicates cPLA_2_γ as a major enzyme involved in phospholipid AA remodeling. Taken together, our results provide new information to understand better the regulatory processes underlying cellular AA availability, which may be used to develop valid strategies to manipulate eicosanoid metabolism and signaling pathways in innate immunity and inflammation.

## Abbreviations

AA: arachidonic acid; CoA-IT, coenzyme A-independent transacylase; GC/MS, gas chromatography coupled to mass spectrometry; LC/MS, liquid chromatography coupled to mass spectrometry; LPS, bacterial lipopolysaccharide; cPLA_2_α, group IVA cytosolic phospholipase A_2_α; cPLA_2_γ, group IVC cytosolic phospholipase A_2_γ; iPLA_2_β, group VIA calcium-independent phospholipase A_2_β; sPLA_2_, secreted phospholipase A_2_; PC, choline-containing phospholipids; PE, ethanolamine-containing phospholipids; PI, phosphatidylinositol; PS, phosphatidylserine.
